# Baseline Patient-Reported Health Status Predicts Hospitalization Duration and Cost in Chronic Heart Failure

**DOI:** 10.7759/cureus.94323

**Published:** 2025-10-11

**Authors:** Alexandra Minca, Claudiu Popescu, Dragos Mincă, Amalia Calinoiu, Adina Rusu, Ana Ciobanu, Valeriu Gheorghita, Dana G Mincă

**Affiliations:** 1 Public Health, University of Medicine and Pharmacy "Carol Davila", Bucharest, ROU; 2 Rheumatology, University of Medicine and Pharmacy "Carol Davila", Bucharest, ROU; 3 Rheumatology, Center for Rheumatic Diseases, Bucharest, ROU; 4 Anatomy, University of Medicine and Pharmacy "Carol Davila", Bucharest, ROU; 5 Internal Medicine, Agrippa Ionescu Emergency Clinical Hospital, Bucharest, ROU; 6 Cardiology, University of Medicine and Pharmacy "Carol Davila", Bucharest, ROU; 7 Infectious Disease, Agrippa Ionescu Emergency Clinical Hospital, Bucharest, ROU

**Keywords:** chronic heart failure, cost of hospitalization, kansas city cardiomyopathy questionnaire, patient-reported outcome, quality of life (qol)

## Abstract

This study examined the significance and independence of patient-reported outcomes (PROs) in predicting hospitalization costs and duration among patients with chronic heart failure (CHF). This observational cross-sectional study included all adult patients with a physician-confirmed diagnosis of CHF who were admitted to the cardiology department of a university emergency hospital in Bucharest, Romania, between July and September 2024. Upon admission, patients completed the validated Romanian version of the 12-item Kansas City Cardiomyopathy Questionnaire (KCCQ) and underwent a clinical interview, physical examination, blood sampling, and transthoracic echocardiography. The primary outcomes were total hospitalization cost and length of stay, whereas the KCCQ Overall Summary Score (KCCQ-OSS) served as the primary predictor in generalized linear models adjusted for potential confounders. The study included 171 patients with a mean age of 73.5 years, of whom 55.0% were women. The median total hospitalization cost was €1,513 per patient, and the mean length of stay was 8.7 days. Each 10-point decrease in KCCQ-OSS was independently associated with a 9.5% increase in expected hospitalization duration, whereas each 10-point increase in KCCQ-OSS was independently associated with a 5.1% increase in expected hospitalization cost, likely reflecting elective procedures in healthier patients. The latter finding likely reflects that patients with better baseline health status were more often selected for elective or interventional procedures, which increased costs despite shorter stays. These results demonstrate that the KCCQ-OSS is an independent predictor of both hospitalization cost and duration in CHF, within the limits of the study (single-center, small sample). Incorporating KCCQ assessment into routine practice may enable earlier identification of high-risk, high-cost patients, inform resource allocation, and enhance patient-centered, value-based management strategies for CHF.

## Introduction

Chronic heart failure (CHF) poses a substantial and growing economic burden on healthcare systems worldwide. In Central and Eastern Europe, the prevalence of heart failure (HF) ranges from 1.6% to 4.7%, with Romania and Slovenia reporting the highest rates, approaching 4.7%. Incidence varies between 3.1 and 6.0 per 1,000 person-years [1]. Romania also records the highest HF-related hospitalization rate in the region, which is more than double the European Society of Cardiology (ESC) median. Cardiovascular mortality remains disproportionately high in Romania, reaching more than twice the average prevalence in the European Union. Data from the Romanian Acute Heart Failure Syndromes (RO-AHFS) national registry [2] reported an in-hospital all-cause mortality of 7.7%, underscoring the severity of presentations and suboptimal rates of evidence-based therapy at discharge. These figures highlight the substantial HF burden in Romania and the wider Eastern European region, as well as the structural and therapeutic gaps that may contribute to worse outcomes compared to Western Europe.

As a leading cause of hospitalization among older adults, CHF accounts for a disproportionate share of healthcare expenditures relative to its prevalence. The economic impact is multifactorial, encompassing direct costs such as inpatient care, outpatient visits, medications, and device therapies, as well as indirect costs related to lost productivity, disability, and informal caregiving. Hospitalizations, in particular, represent the largest single contributor to direct costs, often driven by frequent readmissions and the need for intensive management of decompensated episodes. In the United States alone, the total annual cost of HF is projected to double by 2030 due to aging populations and increasing prevalence [3]. European health systems report similar financial pressures [4]. Importantly, the burden of CHF is not limited to healthcare systems but extends to patients and families through out-of-pocket expenses [5] and diminished quality of life. Efforts to mitigate these costs have increasingly focused on early identification, optimization of guideline-directed medical therapy, and prevention of hospital admissions. Understanding the drivers of cost in CHF, particularly those modifiable through clinical care or self-management, remains critical for developing sustainable healthcare models in an era of rising chronic disease burden.

Patient-reported outcomes (PROs) such as health-related quality of life have also emerged as predictors of healthcare utilization and cost, highlighting the importance of integrating patient perspectives into cost-containment strategies. PROs have also become increasingly recognized as essential tools in the management of CHF [6-8], providing direct insight into patients’ symptoms, functional status, and quality of life. Instruments such as the Kansas City Cardiomyopathy Questionnaire (KCCQ) allow clinicians and researchers to capture the lived experience of HF [9,10], which often cannot be fully assessed through objective measures alone. PROs complement clinical data by revealing limitations in daily activities, psychosocial impacts, and treatment tolerability - all of which are relevant to care planning and prognosis. Beyond their clinical utility, PROs have demonstrated growing value in the economic evaluation of CHF management. Lower PRO scores are consistently associated with increased hospitalization rates, higher healthcare utilization, and poorer outcomes, making them useful predictors of future cost. Several studies have shown that PROs can identify high-risk patients who are likely to incur substantial healthcare costs [11], thereby supporting more targeted interventions, early follow-up, and resource allocation. Moreover, improvements in PROs are increasingly being used as endpoints in cost-effectiveness analyses of therapies [12,13], particularly as healthcare systems shift toward value-based care models. Integrating PROs into routine care may also facilitate shared decision-making, enhance adherence, and improve patient satisfaction - factors that can contribute indirectly to cost reduction. As health systems seek sustainable strategies to manage CHF, PROs offer a unique bridge between clinical effectiveness and economic value, helping to align care with what matters most to patients while optimizing healthcare resources. While the use of PROs to study the economic burden of CHF is abundant in developed countries, such reports from emerging and developing countries are scarce.

Despite evidence from Western populations, no studies have examined the independent predictive value of KCCQ for hospitalization costs in Eastern European CHF cohorts, where healthcare structures and patient characteristics differ substantially. In this context, the current study aims to evaluate the significance and independence of KCCQ in predicting costs of hospitalized CHF cases and their hospitalization duration. While PROs are increasingly recognized for their prognostic value in clinical outcomes, their direct association with healthcare resource utilization and costs remains underexplored, particularly in Eastern European populations. By integrating patient-centered health status measures with detailed cost analyses from a regional cohort, this study aims to bridge a critical knowledge gap and inform both clinical decision-making and health policy.

## Materials and methods

Patients

This observational cross-sectional study included all adult patients with a physician-confirmed diagnosis of CHF who were consecutively admitted through emergency intake to the cardiology department of a university emergency hospital in Bucharest, Romania, between July and September 2024. The recruitment period was limited to three consecutive months to align with the study’s predefined timeline and available resources and to allow consecutive patient inclusion under stable institutional conditions, avoiding major holiday periods and administrative changes that could have influenced admission rates or patient mix. Admissions occurred through the hospital’s routine emergency intake process, in which eligible patients are assigned to available cardiology beds on a first-come, first-served basis, without investigator involvement or pre-selection based on clinical status, prognosis, or other characteristics. Patients with incomplete questionnaire responses, missing CHF characteristics or cost data, and those who died during the admission were excluded. Patients who died during hospitalization were excluded from the analysis to ensure that only completed hospitalizations with full cost accounting and discharge data were analyzed, avoiding cost underestimation associated with truncated admissions. All patients offered written informed consent to participate in the study and for research and publication use of their medical data, and the study was approved by the local ethics committee.

Data collection and measures

Patient demographics (age; sex; dwelling; smoking status), CHF clinical characteristics (ultrasound-estimated left ventricular ejection fraction (LVEF); New York Heart Association (NYHA) functional class [14]), comorbidities (defined with the 10th edition of the International Classification of Diseases codes and used to calculate the Charleson Comorbidity Index (CCI) [15]), laboratory values (N-terminal pro b-type natriuretic peptide (NT-proBNP), normal < 125 pg/mL; serum creatinine, normal < 1.2 mg/dL) and economical characteristics of the hospitalization (total cost, hospitalization duration) were collected from electronic medical records. Upon admission, each patient filled in the Romanian version of the 12-item KCCQ, as kindly provided by the tool’s developers, which is a validated HF-specific PRO measure [9,10]. The KCCQ was selected as the primary PRO measure because it is an HF-specific instrument that has demonstrated high reliability, validity, and responsiveness in diverse HF populations, including those with preserved and reduced ejection fraction, and in both chronic and acute settings. Compared with the generic EQ-5D [16], the KCCQ is more sensitive to HF-specific clinical changes, while offering broader domain coverage and superior interpretability than the MLHFQ [17], with well-established minimal clinically important difference thresholds that support both clinical and research applications [18]. KCCQ was self-administered, and responses were reviewed for completeness immediately after administration; ambiguous or missing answers were clarified with the patient during the same encounter.

Also, each patient underwent clinical interview, clinical examination, and transthoracic echocardiography as part of routine clinical evaluation during the same admission. LVEF was assessed by the clinic’s experienced sonographers in accordance with the guidelines of the American Society of Echocardiography (LVEF was estimated using the biplane Simpson’s method of discs from apical two- and four-chamber views when image quality permitted; in cases of suboptimal image quality, visual estimation by an experienced cardiologist was accepted) [19]. LVEF was used to classify CHF as follows: CHF with reduced LVEF (HFrEF; LVEF ≤ 40%), CHF with mildly reduced LVEF (HFmrEF; LVEF = 41-49%), and LVEF with preserved LVEF (HFpEF; LVEF ≥ 50% with ultrasound-defined left ventricular hypertrophy, left atrium dilatation, or diastolic dysfunction) [20]. Serum creatinine was used to estimate the glomerular filtration rate (eGFR) with the 2009 Chronic Kidney Disease Epidemiology Collaboration (CKD-EPI) equation in order to classify CKD according to eGFR (mL/minute/1.73 m^2^) in stages G1 (≥90), G2 (60-89), G3a (45-59), G3b (30-44), G4 (15-29), and G5 (<15) [21].

Regarding cost, Romanian hospitals issue a hospital bill upon discharge, which represents the cost variable recorded by the study. The amount of the expense bill for each discharged patient includes three components: a) the daily hospital charge per ward/compartment, which is established annually by the hospital and which excludes the value of medicines, medical supplies or services/interventions; b) the number of days of hospitalization completed per discharged case; and c) the value of medicines, including those from national programs, medical supplies, laboratory tests; medical investigations and interventions or maneuvers; food allowance. Total hospitalization costs were extracted from the hospital’s administrative billing database as a single aggregated amount for each admission. Itemized cost components were not available for analysis. For this study, hospitalization cost is reported at an average exchange rate of 5 Romanian Leu per 1 Euro (€). All measures (clinical interview, clinical examination, blood sampling, echocardiography, questionnaire filling) were done within the first two days of the same admission for each patient.

Statistics

Data distribution normality was assessed using descriptive statistics, normality, stem-and-leaf plots, and the Lilliefors corrected Kolmogorov-Smirnov tests. Continuous variables are reported as “mean ± standard deviation” (SD) if normally distributed, or as “median (interquartile range)” (IQR) if non-normally distributed, while nominal variables are reported as “absolute frequency (percentage of group or subgroup)”. Missing data for baseline characteristics were minimal, with <1% missingness for all variables. Given the negligible proportion, no imputation was applied, and analyses were conducted using complete-case data. Group comparisons of hospitalization cost and duration across KCCQ Overall Summary Score (KCCQ-OSS) categories (<25, 25-49, 50-74, ≥75) were performed using Kruskal-Wallis tests. Effect sizes were expressed as epsilon-squared (ε²), representing the proportion of variance in ranked outcomes attributable to group differences. Ninety-five percent confidence intervals (95% CI) for these effect sizes were obtained by nonparametric bootstrap resampling with 1,000 iterations. Total hospitalization cost and hospitalization duration were used as the primary outcomes, while the KCCQ-OSS, a measure ranging from 0 to 100 (higher scores indicating better health status), was used as the primary predictor variable. The association between baseline KCCQ-OSS and the primary outcomes was first assessed using Spearman’s rank correlations. Since the primary outcome measure variables failed normality and homoscedasticity of residuals tests due to their skewness, generalized linear modeling (GLM) with a gamma distribution and log link function was employed to evaluate the independent predictive value of KCCQ-OSS. The gamma distribution is well-suited for continuous, strictly positive, right-skewed data such as healthcare costs [22], as it models variance proportional to the square of the mean and accommodates heteroscedasticity. The log link transforms the linear predictor to a multiplicative scale, facilitating interpretation of regression coefficients as relative (percentage) changes in the outcome. This combination is widely recommended in health economics and health services research for the analysis of cost data [22]. All potential confounders (age, sex, CCI, CKD class, LVEF class, and NYHA class) were entered simultaneously in a single block. Interaction terms were tested to explore potential effect modification by age and sex. Multicollinearity was evaluated using variance inflation factors (VIF), and a threshold of VIF > 5 was considered indicative of significant collinearity. Exponentiated beta coefficients were reported to interpret the effect of predictors as relative changes in expected cost and hospitalization duration. Sensitivity analyses were conducted by stratifying the sample by ejection fraction category (HFrEF vs. HFpEF) and by age tertiles. Given the limited subgroup sizes, these analyses were performed primarily to explore consistency in the direction and magnitude of the association between KCCQ-OSS and hospitalization cost. A formal a priori power calculation was not performed because the study included all consecutive eligible patients admitted during the predefined three-month recruitment period. However, post-hoc estimation indicated that a sample of 171 patients provided adequate statistical power (above 80%) to detect moderate effect sizes (Cohen f² = 0.10) in GLMs with up to six covariates, consistent with the observed magnitude of association between KCCQ-OSS and both hospitalization cost and duration. All tests were performed using IBM SPSS Statistics for Windows (version 26.0, released 2019, IBM Corp., Armonk, NY), and a p-value below 0.05 was considered significant.

## Results

The study included 171 CHF patients with an average age of 73.5 years and a predominance of women (55.0%; Table 1). The sample showed a uniform distribution of NYHA 2-4 classes: 33.3% had NYHA class 2, 33.9% had NYHA class 3, and 32.7% had NYHA class 4. Regarding the type of LVEF, 56.7% were diagnosed with HFpEF, 15.2% with HFmrEF, and 28.1% HFrEF. Regarding economic variables, the sample produced a median total hospitalization cost of 1513 €/patient, with a median daily cost of 260 €/day/patient for a mean hospitalization duration of 8.7 days (Table 1).

**Table 1:** Characteristics of patients, CHF, and hospitalization (n = 171) Normally-distributed data are reported as “mean ± SD”, non-normally-distributed data are reported as “median (IQR)” *Percentage relative to subgroup (CKD). ^#^Cost at an average exchange rate of 5 Romanian Leu per 1 Euro (€). CCI - Charlson Comorbidity Index; CHF - chronic heart failure; CKD - chronic kidney disease; HFm/r/p - heart failure with mildly/reduced/preserved ejection fraction; IQR - interquartile range; NT-proBNP - N-terminal pro b-type natriuretic peptide; NYHA - New York Heart Association classification; SD - standard deviation

Patient characteristics		CHF characteristics	
Women	55.0%	NYHA class 4	32.7%
Age (years)	73.5 ± 9.8	NYHA class 3	33.9%
Urban dwelling	73.1%	NYHA class 2	33.3%
Active smokers	14.0%	HFrEF	28.1%
CCI	6.1 ± 2.1	HFmrEF	15.2%
CKD	73.7%	HFpEF	56.7%
*CKD stage G2	38.1%	NT-proBNP (pg/mL)	2349 (6473)
*CKD stage G3a	29.4%	Hospitalization characteristics	
*CKD stage G3b	19.0%	Total cost (€/patient)^#^	1513 (1341)
*CKD stage G4	7.1%	Daily cost (€/day/patient)^ #^	260 (208)
*CKD stage G5	6.3%	Duration (days)	8.7 ± 7.3

**Figure 1:**
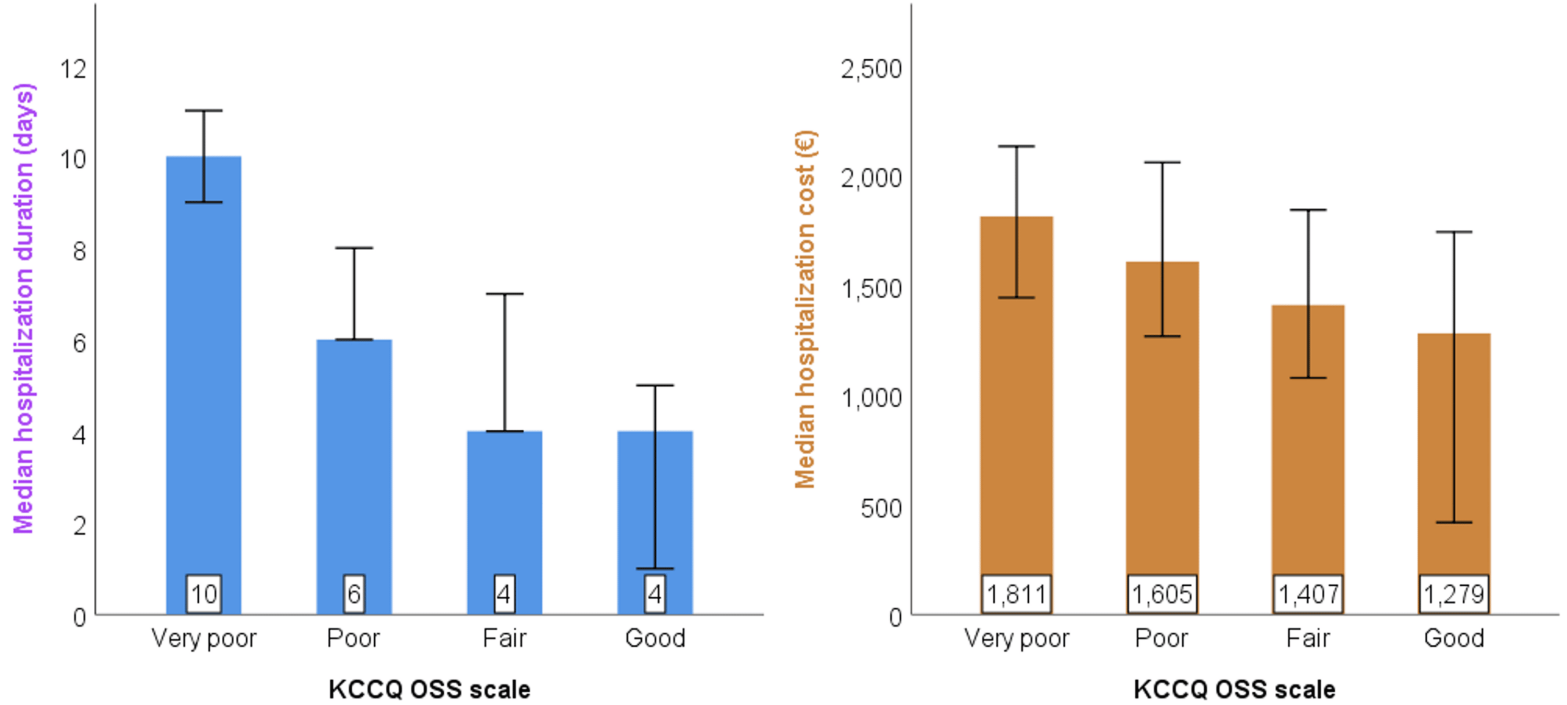
The median variation of hospitalization duration in days (left) and total hospitalization cost per patient in Euro (right) among KCCQ-OSS categories. Both associations were tested with the Kruskal-Wallis test: H = 43; p < 0.001 for hospitalization duration, respectively H = 9; p = 0.029 for hospitalization cost. KCCQ-OSS - Kansas City Cardiomyopathy Questionnaire Overall Summary Score

In terms of PROs (Table 2), 32.7% of patients reported significant physical limitation (very poor KCCQ score), 26.3% of patients reported low symptom stability, 31.6% of patients reported high symptom frequency, 28.1% of patients reported high symptom burden, 26.3% of patients reported low quality of life and 46.2% of patients reported significant social limitation. Despite these reports, 58.5% of patients reported good self-efficacy. When stratifying patients by age (<65 years, n = 28; 65-80 years, n = 101; > 80 years, n = 42), KCCQ-OSS scores differed significantly between groups (p = 0.012; with low effect size: ε2 = 0.064, 95% CI: 0.009-0.522): median KCCQ-OSS values were 43, 40, and 24, respectively, indicating notably lower self-reported health status among patients aged > 80 years compared to younger groups. Generally, median hospitalization duration and median total hospitalization cost increased with each KCCQ-OSS class (Figure 1): compared to patients with good KCCQ-OSS scores (n = 17), patients with very poor KCCQ-OSS scores (n = 66) had significantly higher median hospitalization duration (10 day versus 4 days; p < 0.001; large effect size: ε2 = 0.275, 95% CI: 0.109-0.412) and significantly higher median total hospitalization costs per patient (1811 € versus 1279 €; p = 0.029; small-to-medium effect size: ε2 = 0.072, 95% CI: 0.002-0.102).

**Table 2:** KCCQ scores and categories of CHF patients (n = 171) Scores are reported as “mean ± SD”; KCCQ scores: very poor to poor (KCCQ = 0-24), poor to fair (KCCQ = 25-49), fair to good (KCCQ = 50-74), good to excellent (KCCQ = 75-100). CHF - chronic heart failure; KCCQ - Kansas City Cardiomyopathy Questionnaire; SD - standard deviation

KCCQ category	Mean score	Very poor to poor	Poor to fair	Fair to good	Good to excellent
Physical limitation	39.8 ± 27.7	32.7%	29.8%	21.6%	15.8%
Symptom stability	45.9 ± 36.5	26.3%	17.5%	21.1%	35.1%
Symptom frequency	41.6 ± 29.1	31.6%	30.4%	19.9%	18.1%
Symptom burden	43.9 ± 30.2	28.1%	26.3%	24.0%	21.6%
Total symptom score	42.2 ± 29.4	31.6%	31.0%	18.7%	18.7%
Self-efficacy	72.0 ± 25.5	2.9%	10.5%	28.1%	58.5%
Quality of life	41.3 ± 26.5	26.3%	33.3%	25.7%	14.6%
Social limitation	31.8 ± 30.2	46.2%	22.8%	17.5%	13.5%
Overall summary score	38.8 ± 24.7	38.6%	28.7%	22.8%	9.9%
Clinical summary score	41.0 ± 26.0	28.7%	36.3%	22.2%	12.9%

The GLM model, which examined the association between baseline KCCQ-OSS and hospitalization duration, demonstrated acceptable goodness of fit (deviance/degrees of freedom - df = 0.44; Pearson χ²/df = 0.51) and revealed that baseline KCCQ-OSS was significantly and independently associated with hospitalization duration (Table 3 - model 1). Specifically, each 10-point decrease in KCCQ-OSS was associated with a 9.5% increase in expected hospitalization duration (exp(B) = 0.990; 95% CI: 0.985-0.994; p < 0.001). Other significant predictors included HFrEF, NYHA class, and NT-proBNP level. Similarly, the GLM model, which examined the association between baseline KCCQ-OSS and total hospitalization cost, demonstrated acceptable goodness of fit (deviance/df = 0.37; Pearson χ²/df = 0.68) and revealed that baseline KCCQ-OSS was significantly and independently associated with total hospitalization cost (Table 3 - model 2). Specifically, each 10-point increase in KCCQ-OSS was associated with a 5.1% increase in expected cost (exp(B) = 1.005; 95% CI: 1.000-1.010; p = 0.031). Notably, the direction of the association differed between outcomes: while lower KCCQ-OSS predicted longer hospital stays, higher KCCQ-OSS predicted higher costs. Other significant predictors included HFrEF, NYHA class, and CCI score. Within each model and each subgroup, stratified analyses by ejection fraction category and age tertiles showed that the association between KCCQ-OSS and cost remained directionally consistent with the primary model. However, CIs were wider and some estimates were not statistically significant, likely reflecting limited power due to small subgroup sizes.

**Table 3:** Generalized linear modeling (GLM) for predicting hospitalization duration (model 1) and total hospitalization cost (model 1) using KCCQ-OSS. Each GLM model includes potential confounders (age, sex, CCI, CKD class, LVEF class, and NYHA class) and their interaction terms. p-values are considered significant if < 0.05. df - degrees of freedom; GLM - generalized linear modeling; HD - hospitalization duration; KCCQ-OSS - Kansas City Cardiomyopathy Questionnaire Overall Summary Score; THC - total hospitalization cost; VIF - variance inflation factors

Model performance	Model 1 - HD	Model 2 - THC
Deviance/df	0.44	0.37
Pearson χ^2^/df	0.51	0.68
Likelihood ratio χ^2^	70	54
Df	14	14
p	< 0.001	< 0.001
Predictor performance	KCCQ-OSS	KCCQ-OSS
B	-0.01	0.005
Standard error	0.002	0.002
Wald χ^2^	18.3	4.6
Df	1	1
p	< 0.001	0.031
Exp(B)	0.99	1.005
95% confidence interval	0.985-0.994	1.000-1.010
VIF	1.5	1.5

## Discussion

Main study findings

This study found that baseline patient-reported health status, as measured by the KCCQ, was a significant and independent predictor of total hospitalization cost and of hospitalization duration in patients with CHF: lower KCCQ-OSS scores were associated with higher expected hospitalization duration, even after adjusting for potential confounders. An apparently paradoxical finding was that higher KCCQ-OSS scores were associated with increased total hospitalization costs despite being linked to shorter lengths of stay. This pattern likely reflects differences in case mix and in-hospital management strategies between patients with higher versus lower baseline health status: patients with better functional status at admission may be more suitable candidates for elective or semi-elective interventional procedures (e.g., cardiac resynchronization therapy, device implantation, or coronary revascularization) during the same admission. Such procedures substantially increase direct hospitalization costs while often requiring only a short post-procedure recovery, thereby reducing overall length of stay. In some cases, these admissions may be part of broader care pathways in which inpatient services are integrated with subsequent outpatient follow-up or staged procedures. Thus, a portion of the costs observed in higher KCCQ patients may represent a potential resource allocation towards interventions in outpatient clinics. In contrast, patients with lower KCCQ-OSS scores, reflecting more severe symptoms and poorer functional status, are more likely to require prolonged medical management for decompensated HF, which extends hospitalization duration but does not necessarily result in similarly elevated procedural costs. This interpretation, while speculative in the absence of detailed cost component data, underscores the importance of evaluating both the type and purpose of admissions when interpreting cost associations in CHF populations.

Age-stratified analyses revealed a significant decline in KCCQ-OSS with increasing age, with the lowest scores observed in patients over 80 years. This pattern likely reflects the cumulative impact of comorbidities, frailty, and reduced functional reserve in older patients, as well as possible differences in social support and health literacy. Lower health status scores in the oldest group may indicate higher vulnerability to adverse outcomes and greater resource needs, underscoring the importance of tailored management strategies, including comprehensive geriatric assessment, early rehabilitation, and enhanced outpatient follow-up for elderly CHF patients. In stratified analyses, the association between KCCQ-OSS and hospitalization costs was directionally consistent in both the HFrEF and HFpEF subgroups; however, statistical significance was not uniformly reached. This likely reflects limited statistical power due to smaller sample sizes in each subgroup, as well as differences in clinical characteristics, comorbidity burden, and treatment patterns that may influence both PRO scores and cost profiles, which may introduce greater variability in hospitalization costs and attenuate the strength of association. Similarly, when stratifying by age tertiles, effect directions were preserved, but several estimates did not meet statistical significance thresholds. Smaller effective sample sizes and increased variability in outcomes within age groups likely contributed to wider CIs. Additionally, differences in functional reserve, socioeconomic context, and access to outpatient follow-up across age groups may have introduced heterogeneity in both KCCQ-OSS and cost outcomes, further reducing statistical precision.

The findings suggest that patients’ subjective assessment of their symptoms and functional limitations carries valuable prognostic information beyond traditional clinical metrics. The association between poorer health-related quality of life and increased hospitalization cost and duration highlights the potential utility of integrating PROs into cost prediction and risk stratification models in CHF care. Incorporating PROs such as the KCCQ into routine CHF management could help identify high-cost, high-risk patients earlier, allowing for proactive intervention. In a value-based care context, PROs could offer a cost-effective, patient-centered means of stratifying risk and tailoring resource allocation. In this study, a substantial proportion of patients - ranging from one-fifth to one-third (Table 2) - reported very poor scores (typically defined as < 25) across multiple KCCQ domains, including physical limitation, symptom stability, symptom frequency, symptom burden, quality of life, and social limitation. This finding highlights the considerable symptom burden and functional impairment experienced by a large segment of the CHF population. These low scores reflect the profound impact of CHF on daily life and underscore the importance of addressing not just clinical measures (e.g., NYHA class, ejection fraction, NT-proBNP), but also the patient’s subjective experience.

Interestingly, the self-efficacy domain showed a distinct pattern, with 58.5% of patients reporting good confidence in managing their condition despite generally poor overall health status and symptom burden. This apparent disconnect may reflect several mechanisms. First, self-efficacy represents a subjective sense of control rather than an objective indicator of disease stability (patients may feel capable of adhering to treatment or recognizing symptoms even when their functional capacity is severely limited). Second, cultural and psychosocial factors could influence self-perception, with some patients expressing confidence as a coping or adaptive mechanism despite worsening physical health. Finally, this discrepancy may indicate that conventional education or self-management programs effectively enhance confidence but do not necessarily translate into improved symptom control or quality of life. From a clinical perspective, this finding underscores the need for more nuanced patient education and support strategies. Interventions should not only reinforce self-management behaviors but also integrate realistic goal setting, psychological assessment, and multidisciplinary follow-up to align perceived self-efficacy with actual clinical outcomes. Addressing this mismatch could improve both patient insight and the effectiveness of long-term CHF management.

Comparison with literature

Our findings that lower KCCQ-OSS scores are associated with longer lengths of hospitalization align with existing literature emphasizing the prognostic value of PROs in CHF management [23]. In a seminal study by Chan et al.,patients with the poorest health status (KCCQ score <25) incurred approximately $9,000 more in 12-month healthcare costs compared to those with better scores, underscoring the economic implications of diminished patient-reported health status [24]. Further supporting this, Dai et al. demonstrated that lower KCCQ scores obtained prior to hospital discharge were significantly associated with higher 30-day readmission rates in CHF patients. The study highlighted that each 25-point decrease in KCCQ score corresponded to a substantial increase in readmission risk, suggesting that KCCQ can serve as a valuable tool for identifying patients at elevated risk for rehospitalization [25]. Additionally, research by Sauser et al. indicated that KCCQ scores are sensitive to clinical changes during hospitalization and can reflect improvements or deteriorations in patient status, which may correlate with hospitalization duration [26]. Collectively, these studies reinforce the utility of KCCQ-OSS as a predictive measure for both clinical outcomes and healthcare resource utilization in CHF, aligning with our findings that lower KCCQ scores are indicative of higher hospitalization costs and extended lengths of hospital stay. Already, using tools like KCCQ, studies are investigating alternative care models, such as early discharge to clinic-based therapy of patients presenting with decompensated CHF [27] and early palliative care [28].

Study strengths and limitations

The study has several methodological and contextual strengths. First, it represents one of the few analyses to link PROs with real-world cost data in CHF within an Eastern European healthcare system, addressing a notable geographic evidence gap. Second, the statistical approach (GLM with a gamma distribution and log link) was specifically chosen to accommodate the non-normal, right-skewed distribution of cost data and ensure appropriate inference. Third, the models incorporated a comprehensive set of demographic and clinical covariates, reducing residual confounding and enhancing the robustness of the observed associations. Fourth, effect sizes and CIs were reported transparently for all models, improving interpretability and reproducibility. The identification and interpretation of the paradoxical relationship between higher KCCQ scores and increased costs provide a novel and clinically meaningful insight into the complexity of CHF care. Finally, the use of consecutive admissions and the exceptionally low proportion of missing data (< 1%) support the internal validity of the findings and minimize selection bias.

However, the study has several limitations:

a) A modest sample size was analyzed, which may have limited the power of subgroup analyses. Also, the relatively short data collection period restricted the sample size and may limit the generalizability of the findings to other seasons or to longer-term patterns of CHF admissions and costs. While the study cohort reflects the typical case mix and cost distribution observed in our institution during the study window, future research with extended data collection across multiple time periods and institutions would be valuable to confirm and strengthen the robustness of these results.

b) The Romanian version of the KCCQ has not undergone formal cultural adaptation and psychometric validation. Nevertheless, the instrument has been used in previous research on Romanian CHF patients [29,30] and was well understood by participants in our cohort, supporting its feasibility for use in this setting.

c) Cost data were derived from aggregated hospital billing records, and the lack of itemized data limits the ability to identify specific drivers of hospitalization costs and may constrain the generalizability of our findings to settings with different reimbursement structures.

d) Readmission rates, which could provide additional insight into the prognostic utility of KCCQ-OSS, were not available for this cohort, as patient follow-up beyond discharge was not systematically captured.

e) While cost was modeled using GLM to address skewness, unmeasured confounding and variation in institutional billing practices may influence estimates. Also, lack of data on other potential confounders, such as previous medication use, hospitalization history, socioeconomic status, and alcohol use, may impact the results.

Future studies with larger, multi-center cohorts are needed to validate these findings and to explore whether interventions that improve KCCQ scores can also reduce downstream costs. Integration of PROs into electronic health records and cost prediction algorithms should be a focus of ongoing health system innovation.

## Conclusions

This exploratory study provides novel evidence from an Eastern European cohort that patient-reported health status, as measured by the KCCQ-OSS, independently predicts hospitalization duration and cost in CHF. The opposite directional effects observed (longer stays among patients with poorer health status and higher costs among those with better baseline function) highlight distinct patterns of healthcare utilization driven by clinical severity and procedure-oriented admissions. These findings underscore the practical value of incorporating PROs into hospital-based decision-making to identify patient subgroups requiring either intensified medical management or planned resource-intensive interventions. Integrating such measures into routine care pathways could support more efficient allocation of healthcare resources and strengthen patient-centered management strategies in CHF. Future research should examine whether KCCQ-guided interventions can reduce both symptom burden and healthcare utilization in this population. These findings should be regarded as hypothesis-generating, highlighting the potential of PROs such as the KCCQ to inform both clinical management and economic evaluation in CHF, pending confirmation in larger and more diverse populations.
